# Dental caries prevalence in relation to the cardiovascular diseases: cross-sectional findings from the Iranian kurdish population

**DOI:** 10.1186/s12903-024-04280-z

**Published:** 2024-04-29

**Authors:** Zahra Ghanbari, Yousef Moradi, Negin samiee, Farhad Moradpour

**Affiliations:** 1 Department of oral and maxillofacial medicine, School of Dentistry, Kurdistan University of Medical Sciences, Sanandaj, Iran; 2 Department of Epidemiology and Biostatistics, Faculty of Medicine, Kurdistan University of Medical Sciences, Sanandaj, Iran; 3 Social Determinants of Health Research Center, Research Institute for Health Development, University of Medical Sciences, Kurdistan, Sanandaj, Iran

**Keywords:** Prevalence, DMFT index, Cardiovascular disease, Kurdish people

## Abstract

**Objective:**

Oral and dental health (ODH) is a significant public health concern globally, affecting billions of people. This cross-sectional study aimed to examine the prevalence of dental caries using the DMFT index and investigate its relationship with cardiovascular diseases in the Iranian Kurdish population.

**Methods:**

A total of 3,996 individuals aged 35–70 years were included from the Dehgolan prospective cohort study (DehPCS). Clinical examinations were performed to assess decayed (DT), missing (MT) and filled (FT) teeth (DMFT index). Data on demographics, socioeconomic status, medical history, oral hygiene practices were also collected. Cardiovascular disease (CVD) history was self-reported via questionnaires assessing heart disease, heart attack and stroke. Logistic regression was used to assess associations between oral health indicators and self-reported CVD, adjusting for potential confounders.

**Results:**

The mean DMFT score was 18.18 ± 19, indicating a high oral disease burden. Specifically, 60.04% had DT, 37.82% had more than 16 MT, and 38.83% had FT. Only 13.21% reported flossing regularly and 43.17% brushed less than daily, showing suboptimal oral hygiene. The overall CVD prevalence was 9.21%. Individuals with high DMFT (≥ 14) scores had approximately two times higher CVD prevalence than those with low DMFT. DMFT decreased by 10.23% with increase in education level, and by 5.87% as economic status increased In adjusted analyses, high DMFT scores (OR = 1.5, 95%CI: 1.2–1.9) and MT (OR = 1.5, 95%CI: 1.1–2.1) were associated with 50–150% increased odds of CVD, though associations weakened after adjusting for age. Among men, DMFT remained a significant predictor for CVD after age adjustment, with an odds ratio of 2.37 (95% CI: 1.22–4.60).

**Conclusion:**

This population had substantial oral disease and poor oral hygiene. Higher DMFT scores and MT positively correlated with increased CVD prevalence. Promoting preventive oral care and health education could help reduce dental issues and potentially lower CVD risk. Further research is needed to clarify biological mechanisms linking oral and systemic health.

## Introduction

Oral and dental health (ODH) is widely recognized as a crucial public health concern in numerous countries across the globe. Although the World Health Organization(WHO) has highlighted the importance of ODH and its significant influence on individuals’ daily lives, the lack of focus on ODH and related diseases has had a detrimental effect on over 5.3 billion people globally [1, 2]. Among these, gum inflammation and dental decay are considered the most prevalent diseases related to ODH [3, 4].

Dental decay affects 60 to 90% of students and 100% of adults worldwide, and furthermore, approximately 30% of individuals aged 65 to 74 globally have no natural teeth [5]. Decayed, missing, filled teeth (DMFT index) is the most commonly used scoring system for assessing ODH on a global scale. This index reflects an individual’s oral health status throughout their lifetime, based on their experience of decay [6, 7]. The interplay between ODH and the overall health of other organs, including the cardiovascular system, has been a subject of attention for many years. Dental issues such as dental caries follow a chronic process and, without proper hygiene and a healthy diet, can negatively impact an individual’s health, including their cardiovascular well-being [8]. Advanced/severe stage dental caries was significantly associated with an increase in coronary heart disease (CHD) risk among middle-aged individuals [9].

Moreover, oral and dental diseases share common risk factors with cardiovascular diseases, which are multifactorial in nature, influenced by lifestyle choices, diet, physical activity, tobacco use, and more [10]. Studies have shown that oral microbiota, in addition to these risk factors, can contribute to the incidence of cardiovascular diseases [11].

Despite the overall decline in global mortality rates, cardiovascular diseases stemming from oral and dental diseases continue to be recognized as the leading cause of death and morbidity worldwide. These diseases are responsible for approximately 70 to 80% of deaths and chronic illnesses in both developing and developed countries, accounting for 4 million deaths annually in Europe [12].

While previous studies have primarily focused on clarifying the association between cardiovascular diseases and periodontitis among oral diseases [13], less attention has been given to investigating the relationship between cardiovascular diseases and other dental problems, such as dental caries, filled teeth, and missing teeth [7]. Therefore, considering the paramount importance of ODH and its significant impact on maintaining cardiovascular health, conducting research in this area is imperative. The objective of this study is to assess the prevalence of the DMFT index and explore its association with cardiovascular diseases using data from the Dehgolan prospective cohort study (DehPCS).

## Materials and methods

### Study Design and Population

The present study is based on data collected during the initial phase of the DehPCS, which is a part of the Prospective Epidemiological Research Study in Iran (PERSIAN). DehPCS aims to investigate the incidence, prevalence, mortality, and risk factors of non-communicable diseases among 3,996 study participants aged 35–70 who are permanent residents of Dehgolan, Iran [14]. The study gathered information on demographic characteristics, socio-economic status, and medical history. Excluded from the study were individuals who encountered communication challenges due to conditions like blindness, deafness, and severe mental and psychological illnesses. Additionally, individuals who were excluded included those with tooth loss resulting from factors other than decay, such as trauma and periodontal diseases. The study received approval from the Ethics Committee of Kurdistan University of Medical Sciences under the code of IR.MUK.REC.1402.044.

### Data collection and measurement

Data collection involved face-to-face interviews with individuals. Systematic clinical examinations were performed by tow well-trained nurses according to the WHO criteria to determine the DMFT index scores of participants, providing an objective assessment of caries experience for analysis in relation to cardiovascular health history.

The questionnaire collected information on demographic characteristics, wealth index, body mass index (BMI), personal habits such as smoking and alcohol consumption frequency, dental hygiene practices including dental floss and toothbrush usage, as well as medical history and medication use. The wealth index, which is a composite measure of a household’s living standard, was assessed through a survey conducted at both the household and individual levels. BMI was calculated using a weight (kg)/ height^2^ formula, categorizing individuals into three groups: normal weight (≤ 24.9), overweight (25-29.9), and obese (≥ 30). Smoking status was classified into three groups: non-smoker (an individual who has smoked less than 100 cigarettes in their lifetime), smoker (an individual who has smoked more than 100 cigarettes in their lifetime, ex-smoker (smokers who have not smoke during past 30 days). Alcohol consumption was categorized as individuals consuming 200 milliliters of beer or 45 milliliters of alcohol at least once a week for a minimum of 6 months. Use of Morphine, Heroin, Methamphetamine, Crack, and Cocaine once a week for at least 6 months was considered as illicit/ illegal drug use. Participants were also questioned about their history of ischemic heart disease (including heart failure, angina, and heart attack).

### Oral and dental health assessment

The clinical examination process involved assessing the 32 permanent teeth using the decayed, missing, and filled teeth (DMFT) index [15]. According to standardized guidelines, teeth such as impacted teeth, congenitally missing teeth, supernumerary teeth, deciduous teeth present in adulthood, and teeth lost due to reasons other than decay (such as trauma and periodontal disease) were excluded from the study. Teeth with decay on one surface and restoration on another surface were classified as decayed teeth. The overall score for an individual’s teeth was determined based on the following criteria: 0 (no decayed, missing, or filled teeth) and ≥ 1 (one or more decayed, missing, or filled teeth). Subsequently, individuals were categorized into two groups: high DMFT (DMFT score ≥ 14) and low DMFT (DMFT score < 14) [16]. Missing teeth were classified into three categories of ≤ 5, 6–15, and ≥ 16 missed teeth [17]. In addition to the clinical examination, a questionnaire regarding brushing and flossing frequency was administered and completed by the participants. Calibration exercises involving blinded re-examination of a subsample of participants preformed to check for consistency in DMFT scoring between interviewers.

### Cardiovascular diseases Assessment

Participants were asked questions regarding the presence or absence of common cardiovascular diseases including heart attack, stroke, congestive heart failure or history of any ischemic heart diseases [18]. If they responded positively, additional information was collected, including age at diagnosis, current treatment under a physician’s care, and details about medication type, dosage, and duration.

### **Statistical analysis**

Descriptive statistics including mean numbers of decayed, missing, filled teeth and DMFT index were reported along with their standard deviations. Prevalence of CVD and dental caries were calculated across different variables based on the standard approach as number of individual with positive criteria in numerator divided by all eligible participants in denominator. T-test and one-way ANOVA were used to compare the mean scores across categories. Chi square test also was used to compare outcome of interest between groups. Inter-rater agreement were investigated by calculating kappa statistics. Both unadjusted and adjusted logistic regression models were fitted to examine the associations between DMFT index/components and CVD, presenting odds ratios and 95% confidence intervals. Model 1 adjusted for gender, education, socioeconomic status, BMI, smoking, alcohol, drug use, sugar intake and oral hygiene behaviors. Model 2 further adjusted for age in addition to the covariates in Model 1. All tests were two-tailed with statistical significance set at p- value < 0.05. Statistical analysis were performed using STATA version 17.

## Results

### Prevalence of DMFT, DT, MT, FT

Table 1 presents the DMFT score over different characteristics of study participants. The mean number of DMF among those with at least one decayed and/or missing and/or filling tooth was about 18 ± 19. Prevalence of DT, MT (> 16), and FT were 60.04% (CI: 58.50-61.55), 37.82% (36.32–39.34), and 38.83% (CI: 37.32–40.36) respectively. Approximately all participants suffered from at least one DMFT including DT (female: 57.14% and Male: 63.78%), MT (female: 96.91% and Male: 95.84%), and FT (female: 40.54% and Male: 36.63%). DMFT decreased by 10.23% with increase in education level, and by 5.87% as economic status increased. Only 13.21% of participants used flossing, 43.17% did not brush their teeth daily, 35.35% brushed 1–2 times and 21.58% brushed 3–4 times a day.

**Table 1:** Mean number of decayed, missing, filled teeth, and DMFT index by sociodemographic and ODH (oral and dental health) variables in DehPCS participants

	DMFT	*P*-value	DT	*P*-value	MT	*P*-value	FT	*P*-value
Overall	18.18		2.42		14.12		1.65	
**Gender**								
Male	18.36 ± 9.05	0.17	2.87 ± 3.82	< 0.001	13.56 ± 10.36	0.37	1.53 ± 2.71	0.012^a^
Female	17.96 ± 9.27		2.06 ± 2.81		14.55 ± 10.62		1.74 ± 2.95	
**Age groups**								
35–45	13.30 ± 7.33	< 0.001	2.65 ± 3.23	< 0.001	8.07 ± 7.16	< 0.001	2.58 ± 3.30	< 0.001^b^
46–60	20.65 ± 8.50		2.50 ± 3.47		17.05 ± 9.98		1.09 ± 2.32	
> 60	27.39 ± 6.39		1.26 ± 2.83		25.91 ± 8.02		0.21 ± 1.21	
**Education levels**								
Illiterate	23.23 ± 8.28	< 0.001	2.06 ± 3.33	< 0.001	20.71 ± 9.88	< 0.001	0.46 ± 1.47	< 0.001^b^
1–5	18.22 ± 8.90		2.70 ± 3.38		14.23 ± 10.10		1.29 ± 2.48	
6–12	14.88 ± 8.29		2.78 ± 3.45		9.85 ± 8.52		2.26 ± 3.19	
university	13.00 ± 7.14		1.90 ± 2.64		7.10 ± 7.00		4.00 ± 3.52	
**Economic status**								
Poor	21.20 ± 9.31	< 0.001	2.62 ± 3.85	0.001	18.4 ± 10.88	< 0.001	0.54 ± 1.64	< 0.001^b^
Middle	18.10 ± 9.07		2.45 ± 3.20		14.35 ± 10.14		1.29 ± 2.42	
Rich	15.33 ± 8.06		2.17 ± 2.9		10.03 ± 8.88		3.13 ± 3.49	
**BMI**								
Normal weight	20.11 ± 9.35	< 0.001	2.46 ± 3.75	< 0.001	16.25 ± 11.17	0.002	1.40 ± 271	< 0.001^b^
Over-weight	17.49 ± 9.05		2.43 ± 3.24		13.15 ± 10.24		1.90 ± 3.05	
Obese	17.64 ± 8.94		2.37 ± 3.06		13.78 ± 10.16		1.49 ± 2.63	
**Smoking**								
No smoker	17.09 ± 8.93	0.875	2.25 ± 2.97	< 0.001	12.94 ± 10.22	0.367	1.90 ± 2.98	< 0.001^b^
Ex-smoker	21.93 ± 8.81		2.47 ± 3.60		18.49 ± 3.60		0.97 ± 2.36	
Smoker	21.81 ± 9.03		3.25 ± 4.51		17.79 ± 10.59		0.77 ± 2.05	
**Use alcohol**								
No	18.00 ± 9.11	< 0.001	2.32 ± 3.20	0.006	13.99 ± 10.53	0.01	1.70 ± 2.88	< 0.001^a^
Yes	19.60 ± 9.36		3.15 ± 4.03		15.17 ± 10.51		1.27 ± 2.55	
**Use drugs**								
No	17.59 ± 9.06	< 0.001	2.30 ± 3.09	0.55	13.53 ± 10.41	< 0.001	1.76 ± 2.92	< 0.001^a^
Yes	22.99 ± 8.45		3.37 ± 4.64		18.87 ± 10.24		0.75 ± 1.99	
**Brushing**								
No	27.71 ± 8.05		2.11 ± 3.76		22.23 ± 9.95	< 0.001	0.37 ± 1.35	< 0.001^b^
1	13.06 ± 6.26	< 0.001	2.36 ± 2.71	< 0.001	7.52 ± 5.49		3.17 ± 3.44	
2	13.12 ± 6.14		3.46 ± 2.84		7.40 ± 5.21		3.25 ± 4.06	
3–4	13.51 ± 6.69		3.10 ± 3.15		8.70 ± 5.82		1.70 ± 2.56	
**Use flossing**								
No	19.02 ± 9.26	< 0.001	0.59 ± 0.49	< 0.001	1.20 ± 0.78	< 0.001	0.33 ± 0.47	< 0.001^a^
Yes	12.70 ± 5.89		0.68 ± 0.47		0.55 ± 0.60		0.78 ± 0.41	
**Sugar**								
No	17.13 ± 9.35	< 0.001	0.60 ± 0.49	0.946	12.98 ± 10.58	< 0.001	0.41 ± 0.49	0.0196^a^
Yes	18.90 ± 8.94		0.60 ± 0.49		14.89 ± 10.40		0.37 ± 0.48	
**CVD history**								
No	17.80 ± 9.07	<0.001	2.47 ± 3.33	< 0.001	13.60 ± 10.35	< 0.001	1.73 ± 2.91	< 0.001^a^
Yes	22.02 ± 9.09		1.86 ± 3.17		19.28 ± 10.77		0.88 ± 2.02	

^a^ t-test ^b^ one-way ANOVA

### CVD history

Cardiovascular disease history affected 366 (9.21%) of all participants. The mean age for those with CVD and Not reported CVD history were 54.56 ± 8.8 and 47.71 ± 8.68 respectively. As shown in Table 2 CVD history was not different among males and females, but increased incrementally with a 19.3 prevalence difference between low and high age groups. Taking oral health behavior such as brushing and flossing are accompanied by a lower proportion of CVD history of 6.11 and 5.55% respectively. CVD proportion in people with high DMFT scores was about 2 times more than low DMFT category. As the number of missing teeth increased, there was a corresponding increase in the prevalence of CVD, with a mean of 4.5 (Cochrane-Armitage p-value for trend < 0.001). Additionally, decayed and filled teeth were found to be associated with a lower proportion of reported CVD, approximately 5%.

**Table 2:** Characteristics and proportion of self-reported cardiovascular diseases across sociodemographic and ODH indicators, in DehPCS participants

	CVD history			*P*-value
	Total%	No%	Yes%	
**Gendered**				
Female	2241	2023 (90.27)	218 (9.73)	0.195
Male	1735	1587 (91.47)	148 (8.53)	
**Age**				
35–45	1773	1715 (96.73)	58 (3.27)	< 0.001
46–60	1720	1521(88.43)	199 (11.57)	
> 60	483	374 (77.43)	109 (22.57)	
**Education**				
Illiterate	1245	1053 (84.58)	192 (15.42)	< 0.001
1–5	1110	1022 (92.07)	88(7.93)	
6–12	1113	1046 (93.98)	67 (6.02)	
university	508	489 (96.26)	19 (3.74)	
**Economic status**				
Poor	133	1248 (92.86)	96 (7.14)	0.001
Middle	1300	1180 (90.77)	120 (9.23)	
Reach	1318	1168 (88.62)	150 (11.38)	
**BMI**				
Normal weight	985	917 (93.10)	68 (6.90)	0.010
Over-weight	1698	1535 (90.40)	163 (9.60)	
Obese	1281	1146 (89.46)	135 (10.54)	
**Smoking**				
No smoker	304	2784 (92.06)	240 (7.94)	< 0.001
Ex-smoker	326	271 (83.13)	55 (16.87)	
Smoker	600	532 (88.67)	68 (11.33)	
**Use alcohol**				
No	3470	3162 (91.12)	308 (8.88)	0.069
Yes	481	426 (88.57)	55 (11.43)	
**Use drugs**				
No	3502	3197 (91.29)	305 (8.71)	0.003
Yes	488	390 (87.05)	58 (12.95)	
**Brushing**				
No	1712	149 (87.32)	217 (12.68)	< 0.001
Yes	2254	2106 (93.43)	148 (6.57)	
**Use flossing**				
No	3442	3100 (90.06)	342 (9.94)	< 0.001
Yes	524	501 (95.61)	23 (4.39)	
**DMFT**				
Low	1439	136 (94.86)	74(5.14)	< 0.001
High	2.527	2236 (88.48)	291 (11.52)	
**Decayed teeth**				
No	1585	1387 (87.51)	198 (12.49)	< 0.001
Yes	2381	2214 (92.99)	167 (7.01)	
**Missing teeth**				
low	1038	990 (95.38)	48(4.62)	< 0.001
moderate	1428	1316 (92.16)	112 (7.84)	
high	1500	1295 (86.33)	205 (13.67)	
**Filling teeth**				
No	2426	2155 (88.83)	271(11.17)	< 0.001
Yes	1540	1446 (93.90)	94 (6.10)	

Chi square test.

### Correlates of CVD

The associations of various correlates and confounding variables with CVD were presented in Table 3. Alongside traditional risk factors like age, BMI, smoking, illicit drug use, and education, the practice of teeth brushing and flossing showed a significant association with lower odds of CVD in the population. The practice of teeth brushing (OR = 0.48, 95% CI: 0.39–0.60) and flossing (OR = 0.42, 95% CI: 0.27–0.64) showed a significant association with lower odds of CVD in the population. Notably, the specific ORs were not reported in Table 3.

Additionally, in the unadjusted analysis, both the DMFT index and missing teeth were positively associated with CVD, while decayed and filled teeth were negatively associated with CVD. However, in the multivariable analysis, after adjusting for several confounders including gender, education, socioeconomic status, BMI, smoking, alcohol use, illicit drug use, and sugar consumption and ODH behaviors (model 1), the DMFT index and high missing teeth were shown to increase the odds of CVD by approximately 50%, with an OR of 1.54 (CI: 1.13–2.09) and 1.58.

(1.03–2.43) respectively. On the other hand, decayed teeth were found to decrease the odds of CVD by 31% with an OR of 0.69 (CI: 0.53–0.90).

Further adjustments for age (model 2) weakened the associations between DMFT index and missing teeth with CVD, although the association remained statistically significant for dental caries OR = 0.75 (CI: 0.57–0.99). Because of significant interaction between gender and DMFT (p-value < 0.05) results were also reported based on gender strata. In the gender-stratified analysis, after performing additional adjustments for age (model 2), most of the associations diminished among women. However, among men, DMFT remained a strong positive predictor 2.37 (CI: 1.22–4.60) for CVD.

**Table 3:** Adjusted and unadjusted logistic regression (odds ratio) of decayed, missing, filled teeth, and DMFT index on cardiovascular diseases

	DMFT (Ref: Low)	Decayed tooth (Ref: No)	Missing tooth (Ref: Low)		Filled tooth (Ref: No)
In all participants			Moderate	High	
**Unadjusted Model**	2.40(1.84–3.12)^c^	0.53(0.4–0.66) ^c^	1.75(1.24–2.49)^b^	3.26(2.36–4.52) ^c^	0.52(0.40–0.66) ^c^
Adjusted Model 1	1.54 (1.13–2.09)^b^	0.69 (0.53–0.90)^b^	1.40 (0.97–2.02)	1.58 (1.03–2.43)^a^	1.09 (0.78–1.52)
Adjusted Model 2	1.16 (0.84–1.59)	0.75 (0.57–0.99)^a^	1.11 (0.76–1.62)	1.00 (0.64–1.58)	1.14 (0.82–1.60)
**In female**					
Adjusted Model 1	1.15 (0.77–1.71)	0.65 (0.46–0.91)^a^	1.33 (0.81–2.18)	1.31 (0.72–2.39)	1.15 (0.75–1.76)
Adjusted Model 2	0.84 (0.49–1.43)	0.73 (0.50–1.06)	1.12 (0.67–1.84)	0.96 (0.51–1.80)	1.19 (0.77–1.84)
**In male**					
Adjusted Model 1	2.40 (1.47–3.91) ^c^	0.67 (0.48–1.02)	1.45 (0.83–2.53)	1.89 (1.02–3.50)^a^	0.99 (0.59–1.70)
Adjusted Model 2	2.37 (1.22–4.60)^b^	0.84 (0.57–1.24)	1.09 (0.61–1.94)	1.04 (0.53–2.04)	1.01 (0.59–1.75)

^a^ *P*-value < 0.05, ^b^ *P*-value < 0.01, ^c^ *P*-value < 0.001

Model 1, OR adjusted for gender, education, socioeconomic, BMI, Smoking, Alcohol use, drug use, Sugar use, brushing, flossing

Model 2, adjusted for variables included in Model1 and age

## Discussion

The results of this study provide important insights into the oral health of the population and its relationship with CVD. Results provide a mean DMFT score of 18.18 including 60.04% DT, 37.82% of high MT, and 38.83% FT. DMFT with an OR of 2.37 presents a moderate effect size on CVD in men. Compared to global data, our DMFT is lower than the global median of 21.9 reported for ages over 60 years in a 2021 systematic review [19]. Studies across Asia have found various DMFT of 21.9 in Turkey [20], 22.5 in China [21], and 14.3 in Vietnam [22]in comparable age groups. Previous studies have reported lower mean DMFT score of 7.8 [23], and 14.8 in the regional and national level respectively [24].The divergent DMF index reported may reflect context-specific population differences. Notably, a recent comparable study in culturally similar Kermanshah, Iran found a near-identical 18.08 DMFT, corroborating socio-cultural impacts on oral health [25].

The high prevalence of untreated caries in our study can be concerning for functional impairment and quality of life. Studies from United States, Malawi [26], Egypt [27] and Pakistan in similar age groups found decay teeth prevalence of 21.5, 49 and 56.6% respectively, lower than our prevalence. High prevalence of missing teeth about 96% in our study is much more the other studies in Malawi 63%, Egypt 76%, likely indicates inadequate preventive dental care and unmet treatment needs in population. This study indicate high level of caries experience according to the WHO [15]. The combination of obstacles relating to diet, oral hygiene, socioeconomics, geography and health literacy have created an environment conducive for extensive dental caries to take hold in this population [28].To our knowledge, only 13% of our participants used dental floss and 43% did not brush daily, indicating suboptimal oral hygiene practices significantly less than other international and national studies in the Malawi, Egypt [27] and Tehran [29] which reported 2.9 and 23 and 19% did not daily brushing their teeth.

The study found that individuals with poor oral health, as indicated by high DMFT scores and missing teeth, had a higher prevalence of CVD. In contrast, decayed and filled teeth were associated with a lower proportion of reported CVD. MT could reflect worse long-term oral health status and cumulative caries burden over many years, capturing the influence of chronic inflammation on systemic health. On the other hand, DT represents more active but potentially treatable disease states. Those with high DT may practice better oral hygiene or have better access to care, reducing inflammatory impacts [30]. We also found that oral health behaviors such as brushing and flossing were associated with a lower proportion of CVD history. This is consistent with previous studies conducted in German [31] and Finnish [32] population. People who regularly use dental floss and toothbrush have lower levels of C-reactive protein (CRP) in their bloodstream. This protein is an indicator of inflammation in the body, and an Increase in Its amount in the body is associated with an increased risk of cardiovascular diseases [33].

The prevalence of CVD in individuals with high DMFT was more than twice as high as those with low DMFT and is consistent with the results of a study conducted in Japan [34]. While the current study did not investigate potential biological mechanisms, prior research has implicated the oral bacterium Streptococcus mutans, commonly associated with dental caries development, as one possibility warranting further study in relation to cardiovascular disease risk [35].

The findings of the present study indicate a positive correlation between the prevalence of heart diseases and the number of missing teeth. This observation aligns with a recently published systematic review that has established a connection between tooth loss and the occurrence of cardiovascular events, including coronary artery disease (CAD), peripheral arterial disease, and an elevated risk of stroke-related mortality [36]. The possible pathways In this mechanism may include a link between chronic systemic inflammation associated with periodontal disease, an Indirect effect on the cardiovascular system through dietary changes resulting from tooth loss, and the loss of normal gum tissue following progressive dental injury, which leads to the accumulation of oral microbes in the depths of the oral tissue. As these microbial accumulations grow and progress, they put the individual at risk for cardiovascular disease [37, 38].

After adjustment for several confounding variables, including gender, education, socioeconomic status, BMI, smoking, alcohol use, illicit drug use, sugar consumption, brushing, flossing (model1) high DMFT index and high MT were associated with a 1.5-fold increase in the chance of CVD. Further adjustment for age (model 2) revealed that age play a major confounding role in the association between oral health and CVD. An interesting finding of our study is interaction between DMFT and gender. So, gender-stratified analysis showed that after adjusting for covariates, there was a significant association between a high DMFT index and self-reported history of CVD among males, but not females [39]. Age plays a significant role in confounding factors for females, primarily due to variations in the timing of reproductive and hormonal changes throughout their lifespan, which in turn influence disease risk. When adjusting for age, a greater portion of the association is accounted for in females [40]. DMFT scoring system as a surrogate for ODH represents past and present dental caries [7]. Studies show that dental caries is the primary driver for tooth extractions and the main factor causing tooth loss when looking at causes in individuals [41]. Given that our study was cross-sectional the high prevalence of missing teeth, even with prevalence of decayed/filled teeth, suggests that extraction may have been prioritized over restoration in the treatment of dental caries. Tooth loss (≥ 8) following decay can lead to changes in dietary patterns, resulting in elevated levels of total cholesterol and triglycerides, as well as the potential for obesity, high blood pressure, and ultimately cardiovascular diseases [11]. On the other hand, inflammation in periodontal pockets following tooth loss can trigger chronic systemic inflammation, thereby increasing blood pressure and the risk of cardiovascular diseases [17, 42]. Although other studies have concluded there is an independent relationship between missing teeth and CVD, there has been no study investigating the DMFT index as a surrogate for lifetime dental caries experience [43–45]. However, our study findings highlight a high DMFT index as a significant factor in the cardiovascular health of older adults.

This study has several strengths. It makes use of recent and representative data from a large sample of the Kurdish population, which has not been done before. Comprehensive list of risk factors was included in the logistic regression model to address residual confounding. However, the limitations inherent in the study design should be considered when interpreting the results. As is typical with cross-sectional designs, while connections can be identified, the timeline of relationships and underlying causation cannot be validated. This work also relied on self-reported information, allowing for possible recall/reporting bias.

### Conclusion

This study found a high burden of oral diseases among Kurds based on a high mean DMFT score, substantial untreated dental caries and missing teeth. Poor oral hygiene practices were observed versus other studies. Poor oral health as indicated by a higher DMFT score would be positively associated with cardiovascular disease. Promoting preventive oral care and oral health education could help reduce preventable dental issues and potentially lower long-term cardiovascular morbidity and mortality. The study emphasizes the importance of integrated oral-overall health approaches and highlights unmet oral health needs, underscoring the need for dental-medical collaborations to advance oral health and cardiovascular well-being through addressing modifiable risk factors.

## Data Availability

The datasets used and/or analyzed during the current study are available from the corresponding author on reasonable request.
